# Supplementary material for: Amyloid-β aggregates activate peripheral monocytes in mild cognitive impairment
Source: Nat Commun. 2024 Feb 9;15:1224. doi: 10.1038/s41467-024-45627-y (PMC10858199; doi:10.1038/s41467-024-45627-y)
Supplement: Supplementary file 1 — Supplementary Information [file 41467_2024_45627_MOESM1_ESM.pdf]

Supplementary Information for

Amyloid-β aggregates activate peripheral monocytes in mild cognitive impairment

Kristian Juul-Madsen, Peter Parbo, Rola Ismail, Peter L. Ovesen, Vanessa Schmidt, Lasse S. Madsen, Jacob Thyrsted, Sarah Gierl, Mihaela Breum, Agnete Larsen, Morten N. Andersen, Marina Romero-Ramos, Christian K. Holm, Gregers R. Andersen, Huaying Zhao, Peter Schuck, Jens V. Nygaard, Duncan S. Sutherland,
Simon F. Eskildsen, Thomas E. Willnow, David J. Brooks, Thomas Vorup-Jensen
Correspondence to: vorup-jensen@biomed.au.dk

This PDF file includes:

Suppl. Figs. 1-14
Suppl. Table 1
Suppl. Methods
Suppl. References

Supplementary Figures

Supplementary Fig. 1

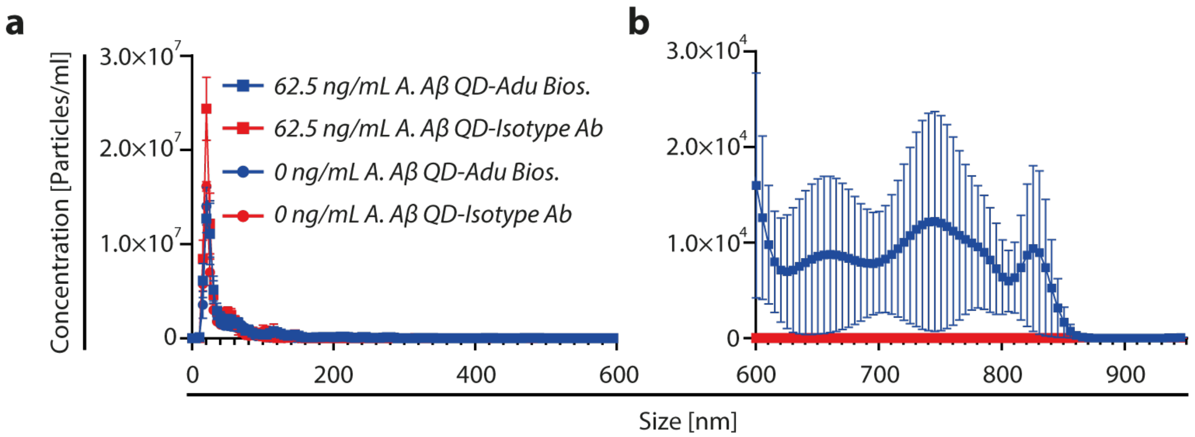

Full size range detection of Aβ spiked into plasma with QD-Adu Bios and QD-isotype control Ab. **a** Size range from 0-600 nm and concentration of detected QD conjugates in plasma spiked with 62.5 ng/ml or 0 ng/ml Aβ. **b** Concentration of particles of detected

Supplementary Fig. 2

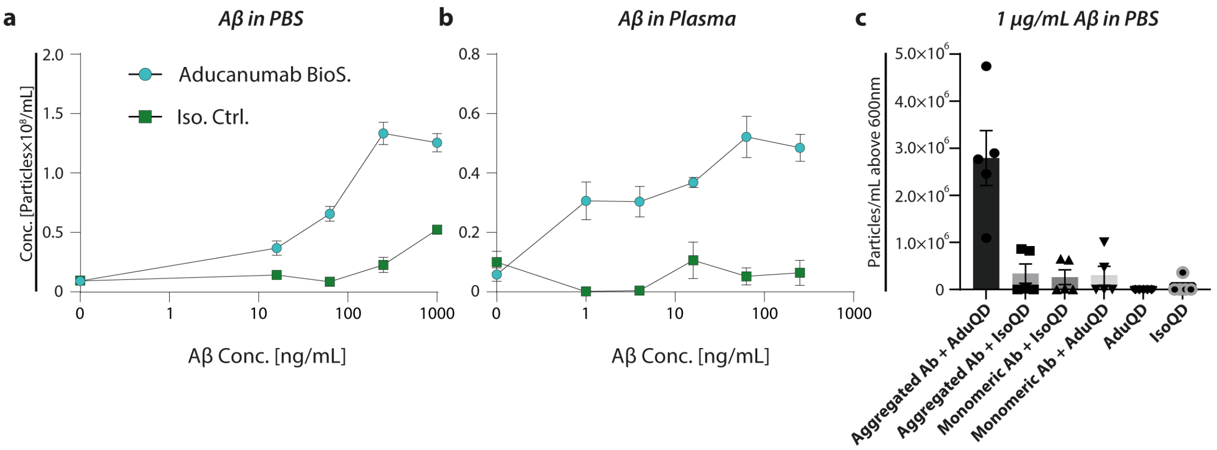

NTA detection Aβ with aducanumab biosimilar. or isotype control in PBS and plasma and of > 600 nm Aβ aggregates in PBS. a Total particles detected with aducanumab bios. and isotype control QD-conjugates in PBS as a function of Aβ concentration. **b** Total particles detected in with Aducanumab bios. and isotype control QD-conjugates in 1:10 dilution of HC plasma as a function of Aβ concentration. **c** Quantifying particles above 600 nm detected in FDM NTA from aducanumab biosimilar (adu) or isotype control Ab-coupled QDs with (-) Aβ, monomeric Aβ, or aggregated Aβ. Error bars indicate SEM.

Supplementary Fig. 3

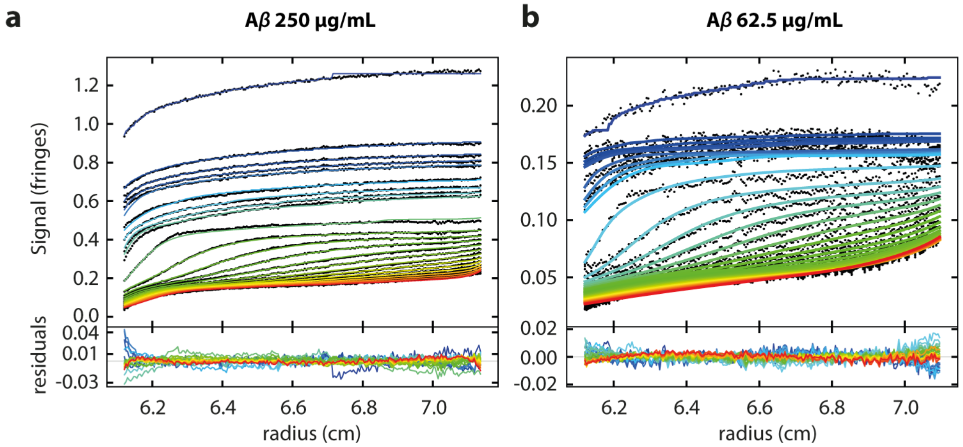

SV-AUC of A-Aβ in PBS 1mM EDTA. a,b Rayleigh interference optical sedimentation boundaries of A-Aβ. Best-fit from the c(s) model at 250 μg/mL (a), and 62.5 μg/mL (b), sedimenting at 3,000, 10,000, and 40,000 rpm. Residuals are shown in the lower panel. Each scan is shown in a color temperature indicating the evolution of time.

Supplementary Fig. 4

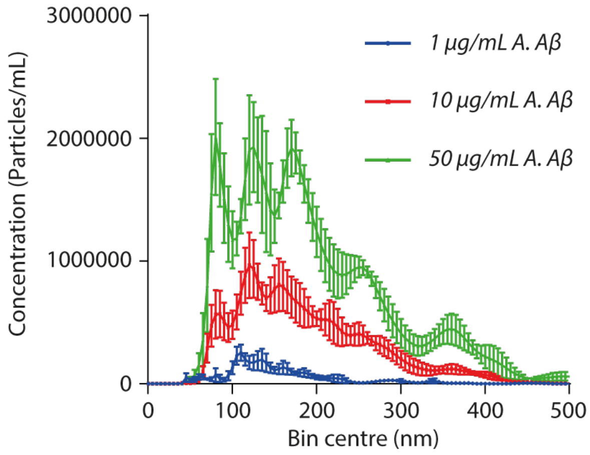

NTA detection Aβ for SV-AUC analysis in scatter detection mode. Size distribution of Aβ 1 μg/mL, 10 μg/mL, and 50 μg/mL in PBS 1mM EDTA. Error bars indicate SEM of 5 technical replicates.

Supplementary Fig. 5

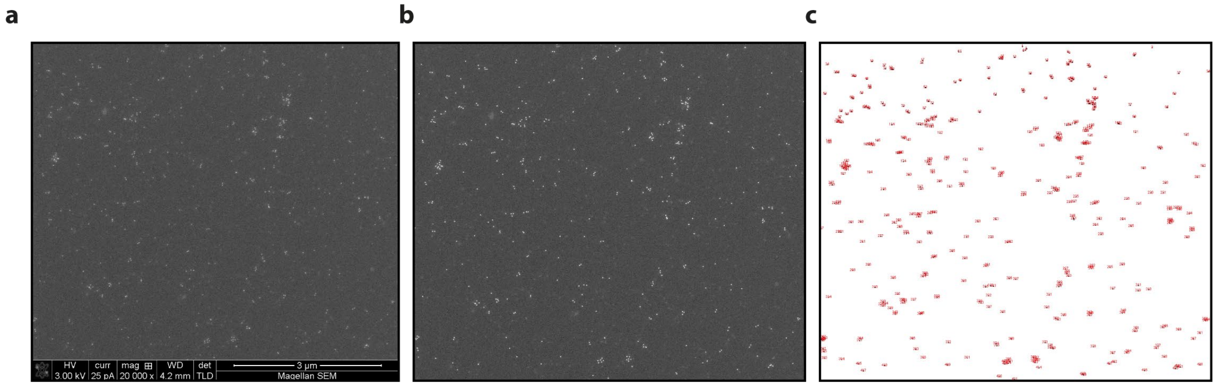

Identification of clustering in A-Aβ incubated with Aducanumab bios or isotype control QDs. a Raw SEM image of a silicon wafer Ti PVD-coated surface treated with aggregated Aβ 5 µg/mL for 30 min and incubated with Aducanumab bios. Ab QD-conjugates. **b** Manual contrast enhancement of the raw image using ImageJ[1]. **c** Counting and positioning of the particles using analyze particles function in ImageJ. Images are representative of 6 technical replicates for each of 3 independent experiments for each condition.

Supplementary Fig. 6

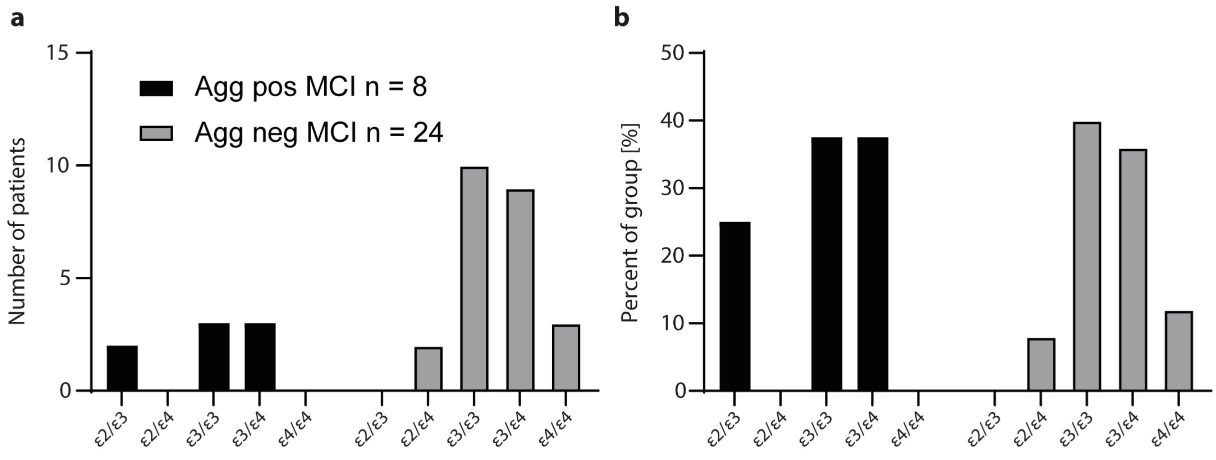

APOE genotype of MCI Agg pos. and neg. patients
a Number of patients in each of the observed APOE genotypes. **b** Percentage of patients in each group.

Supplementary Fig. 7

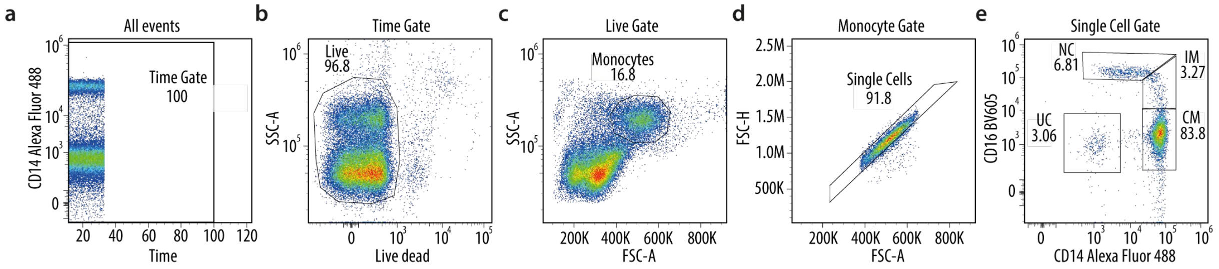

f

Antigen	Fluorochorme	Conc. (ug/mL)
CD14	AF488	8.2
CD16	BV605	4.7
CD18	BV421	1.9
CD163	PE	2.9
KIM127	APC *	17.5

Gating strategy for monocyte subsets from HC and MCI patients. a Staining was verified as constant during analysis. **b** Gating of live cells. **c** gating of monocytes based on FSC/SSC. **d** gating of single cells. **e** Gating of monocyte subsets. Unclassified monocytes (UC) CD14- CD16-, classical monocytes (CM) CD14++ CD16-, intermediate monocytes (IM) CD14++ CD16+, and non-classical monocytes CD14+ CD16++. **f** Table of fluorophores and concentrations used for staining of PBMCs. *Conjugated to APC in-house using Lightning-Link APC conjugation kit (Innova Biosciences, cat. 705-0010). # Antibody produced in hybridoma ATTC CRL-2838 and purified by Genscript, reconstituted to a final concentration of 1 mg/mL.

Supplementary Fig. 8

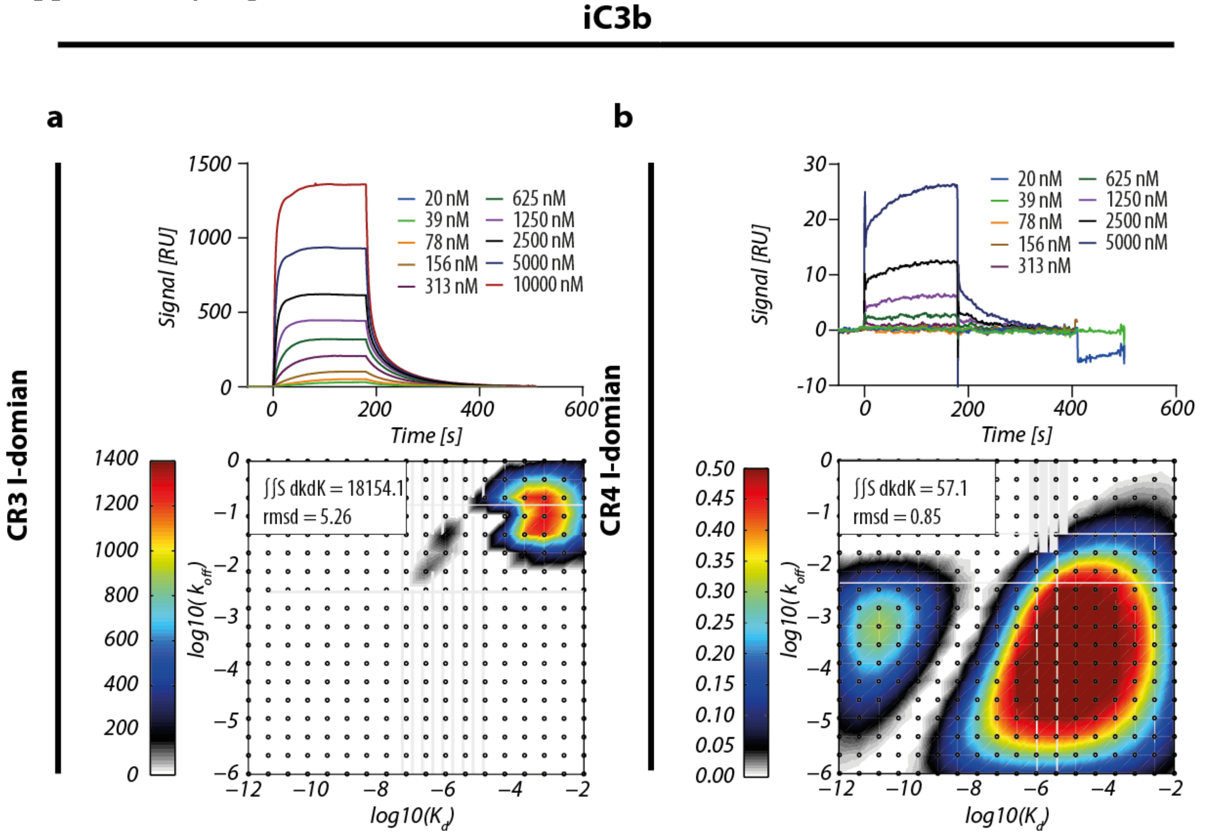

The binding of CR4, but not CR3, to iC3b equals the binding to Aggregated Aβ. a,b SPR analysis of CR3- (a) and CR4-I-domain (b) binding to complement iC3b coupled on the sensorchip. All sensorgrams were fitted using EVILFIT[2] with parameters matching those for the Aβ analyses. In the 2D-fit, the total sum of binding and the RMSD from the fits are indicated showing an error margin below 2% for all experiments. All sensorgrams represent two independent experiments, and 2D kinetics agree with the previously published analysis of CR3 and 4 binding to iC3b[3].

Supplementary Fig. 9

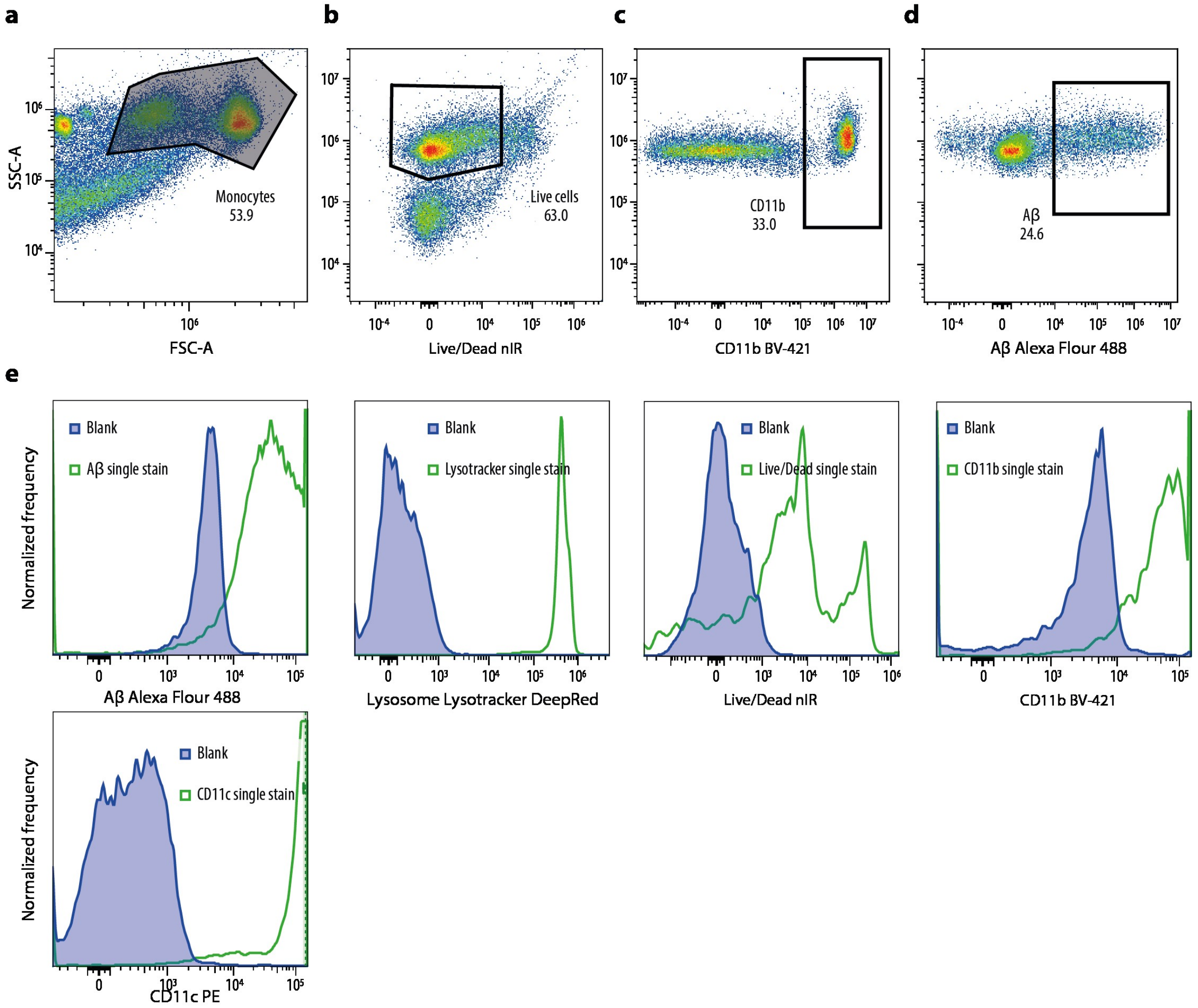

Gating strategy for primary human monocytes for Aβ phagocytosis. a Cells gated on FSC-A/SSC-A **b** Live cells gated based on L/D/SSC-A. **c** CD11b- and **d** CD11c-positive cells. **e** Single stains of Aβ Alexa Flour 488, Lysotracker Deepred, Live/Dead nIR, CD11b BV421, and CD11c PE compared to unstained cells.

Supplementary Fig. 10

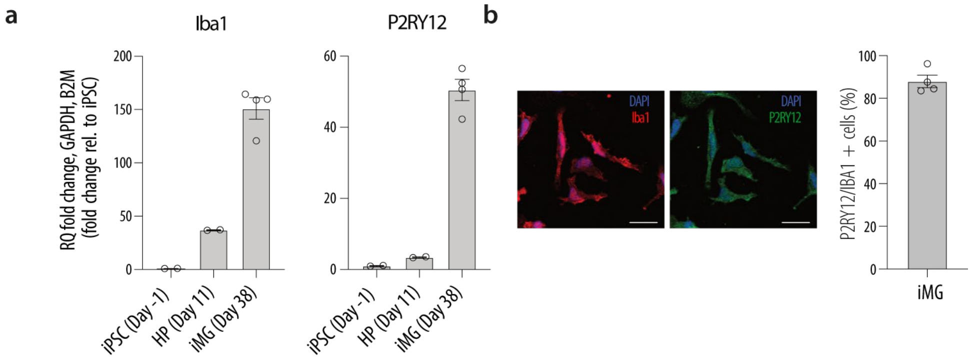

Characterization of iPSC-derived microglia. a qPCR of iMG markers iba1 and P2RY12 at iPSC level, day 11, day 23, and day 38 show increasing expression of a function of iMG maturation. n = 2, 2, and 4 for day -1, day 11 and day 38 respectively. Error bars indicate SD. **b** Mature microglia on coverslips immunostained for DAPI (blue) and microglia-marker Iba1 (red) or P2RY12 (green). The scale bar represents 100 µm. Quantification of cells (DAPI) double positive for Iba1 and P2RY12 were made using ImageJ, n = 4.

Supplementary Fig. 11

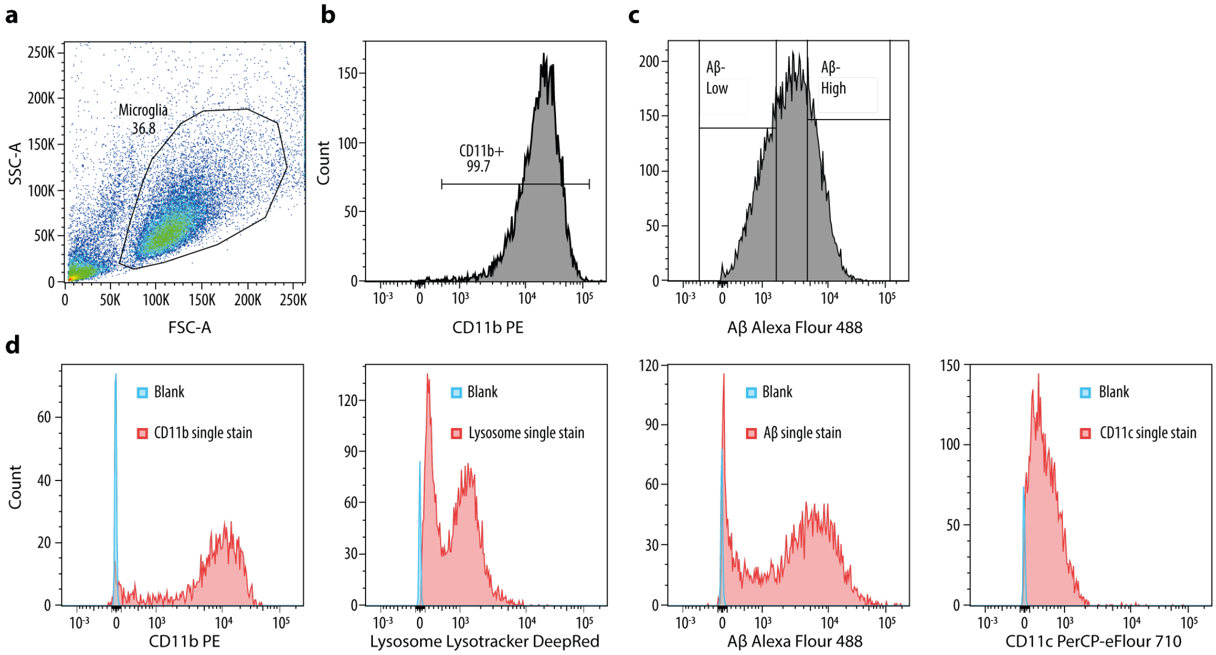

Gating strategy for iPSC-derived microglia for FACS analysis. **a** Cells gated on forward scatter (FSC-A)/side scatter (SSC-A). **b** CD11b-positive cells. **c** Gating of iMG into low and high Aβ uptake for further qPCR analysis. **d** Single stains of CD11b PE, Lysosome Lysotracker (DeepRed), Aβ (Alexa Flour 488), and CD11c (PerCP-eFlour 710) compared to unstained (blank) cells.

Supplementary Fig. 12

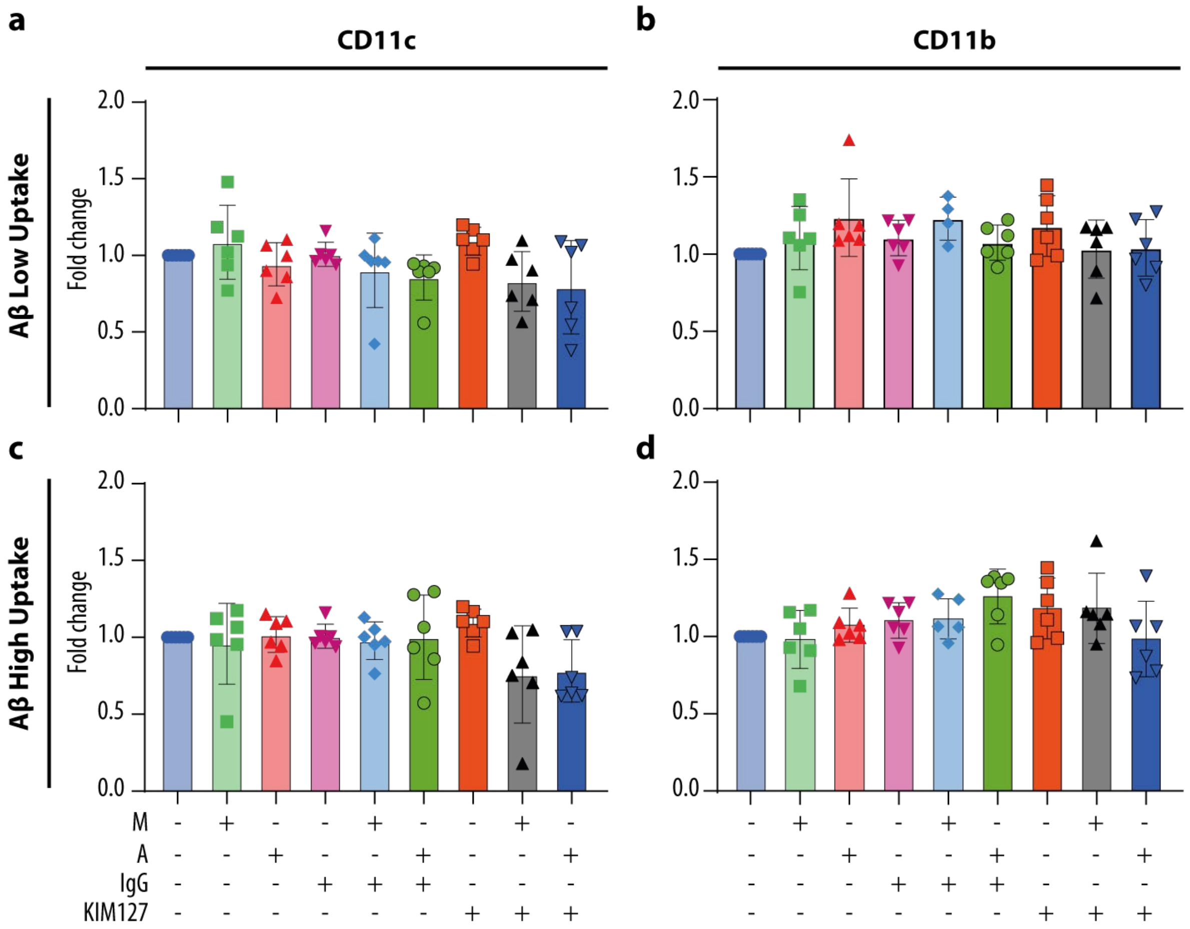

RT-qPCR from FACS sorted microglia with Aβ-low, or Aβ-high uptake with no Ab, IgG control Ab or KIM127 Ab activation. a,b RT-qPCR of FACS-sorted, Aβ-low microglia for CD11c gene (ITGAX) (a) and CD11b (ITGAM) (b) transcription. **c** RT-qPCR of Aβ-high microglia for CD11c (c) and CD11b (d) gene transcription. n = 6. Error bars represent SD.

Supplementary Fig. 13

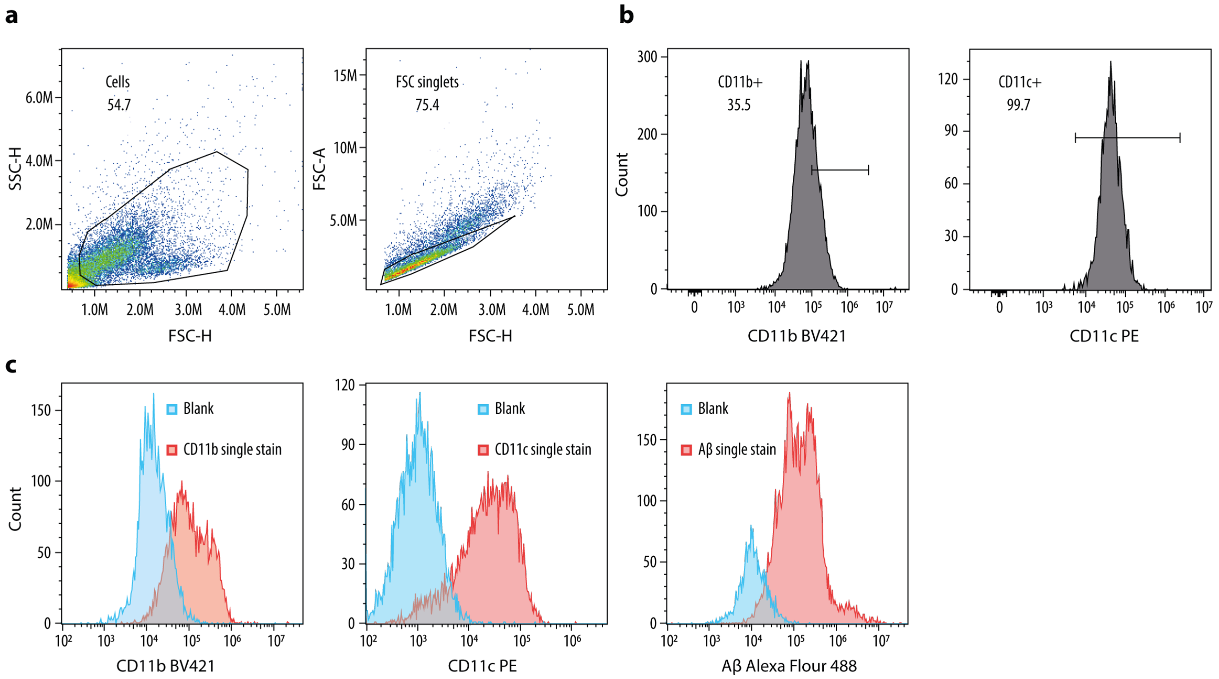

Gating strategy for iPSC-derived microglia for 3.9 Ab inhibition. a Cells gated on FSC-H/SSC-H and single cells gated based on FSC-A/FSC-H. **b** CD11b- and CD11c-positive cells. **c** Single stains of CD11b BV421, CD11c PE and, Aβ Alexa Flour 488 compared to unstained cells.

Supplementary Fig. 14

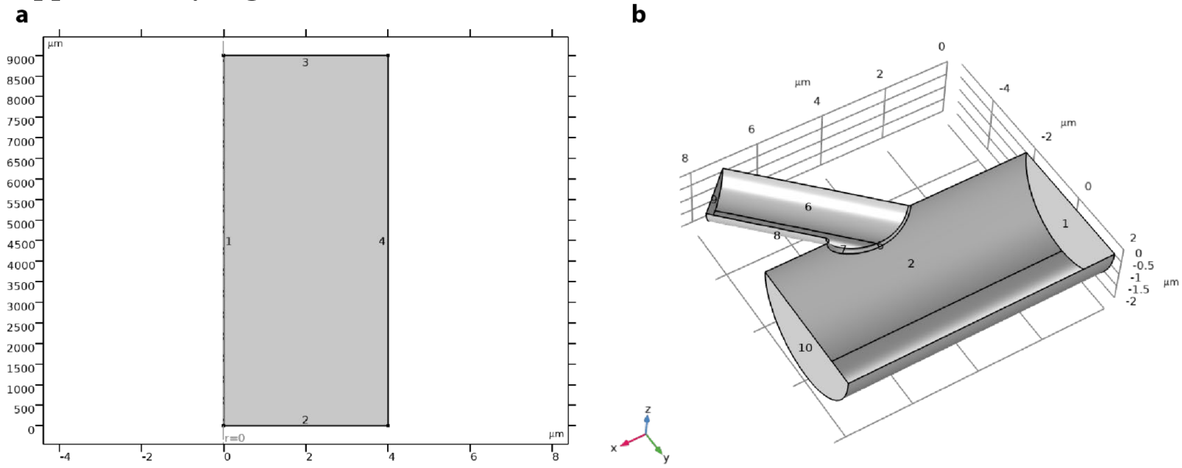

Geometries of two model blood vessels investigated in Problems A and B. a,b Problem A is a 2D rotational symmetric 9 mm long and 4 µm in radius wide geometry (a).Problem B is a 3D 8 µm long and 4 µm in radius wide vessel with a branch at an angle of 60° (b). Symmetry in the xy-plane is applied to reduce computational costs. Numbers refer to the description of boundary conditions applied as indicated in Suppl. Methods.

Supplementary Table 1

	MCI AGG [-] N = 28	MCI AGG [+] N = 10	HC N = 17
AGE, YEARS, MEAN ± SD [RANGE]	*72.9 ± 6.7 [59-85]*	*70.3 ± 10.5 [52-83]*	*73.3 ± 5.0 [65-82]*
WEIGHT, KG, MEAN ± SD [RANGE]	*76.3 ± 13.3 [42-109]*	*77.5 ± 20.4 [45-105]*	*66.8 ± 13.3 [53-79]*
SEX, MALES, N (%)	*21 (75)*	*4 (40)*	*8 (47)*
SUBJECTS USING NSAID, N (%)	*10 (36)*	*2 (20)*	*3 (18)*
MMSE SCORE, MEDIAN [RANGE]	*26.5 [17-30]*	*26.5 [21-29]*	*28.0 [27-30]*
CRD SUM OF BOXES, MEDIAN [RANGE]	*3 [0-9]*	*1.75 [0-7]*	*0 [0-1]*
AMYLOID LEVEL (AB+/AB-)	*18/8*[*]*	*2/8*	*2/7*[†]*

*2 missing ^{11}C-PiB scan

[†]10 missing ^{11}C-PiB scan

Participant characterization
Data are presented as mean ± SD, number of subjects (%) or medians. MMSE = Mini-Mental State Examination; NSAID = non-steroidal anti-inflammatory drug; CDR = Clinical dementia rating.

Supplementary Methods

Hydrodynamic simulation of particle movement in blood vessel

The stationary fluid dynamic problem described by the Navier-Stokes equations is solved within the geometry as shown in Suppl. Fig. 15 by using thermophysical properties as described in Methods. The time-dependent particle tracing is completed over 3 ms in steps as chosen by a standard solver configuration.

	**Problem A**	**Problem B**
Fluid dynamics problem	$\rho(\vec{u}\cdot\nabla)\vec{u} = \nabla\cdot[-pI + K]$<br>$\rho\nabla\cdot\vec{u} = 0$	
Symmetry conditions	along line 1	along xy-plane at z = 0
Initial conditions	$p = 0$ and $\vec{u} = 0$ in the whole domain	
Inlet conditions	developed flow @ line 2	developed flow @ surface 1
Outlet conditions	$p = 0$ @ line 3	$p = 0$ @ surface 9<br>$p = 40\ [Pa]$ @ surface 10
Boundary conditions	$\vec{u}\cdot\vec{n} = 0$ @ line 4	$\vec{u}\cdot\vec{n} = 0$ @ remaining surfaces

	**Problem A**	**Problem B**
Particle tracing problem		
Symmetry conditions	along line 1	along xy-plane at z = 0
Initial conditions	$p(x,y,z)$ and $\vec{u}(x,y,z)$ fields in the whole domain as obtained from solving the fluid dynamic problem	
Inlet conditions	20000 particles @ line 2	20000 particles @ surface 1
Outlet conditions	disappear	disappear
Boundary conditions	freeze @ remaining surfaces $E_p = \frac{1}{2}m_p\lvert\vec{u}\rvert^2$	
Lift force (all domains)	$F_L = \frac{\rho r_p^4}{D^2}\beta(\beta G_1(s) + \gamma G_2(s))\vec{n}$<br>$\beta = \left\lvert D(\vec{n}\cdot\nabla)\vec{u}_{\parallel}\right\rvert \quad \gamma = \left\lvert\frac{D^2}{2}(\vec{n}\cdot\nabla)^2\vec{u}_{\parallel}\right\rvert \quad \vec{u}_{\parallel} = (I - \vec{n}\otimes\vec{n})\vec{u}$	
Drag force (all domains)	$F_D = \frac{1}{\tau_p}m_p(\vec{u} - \vec{v_p})$ and $\tau_p = \frac{\rho_p d_p^2}{18\mu}$	

All used as implemented in Comsol version 6.1. For further explanations and definitions, we refer to their documentation and the scientific literature cited within.

References

1. Schneider, C.A., Rasband, W.S. & Eliceiri, K.W. NIH Image to ImageJ: 25 years of image analysis. *Nat Methods* **9**, 671-675 (2012).

2. Gorshkova, II, Svitel, J., Razjouyan, F. & Schuck, P. Bayesian analysis of heterogeneity in the distribution of binding properties of immobilized surface sites. *Langmuir* **24**, 11577-11586 (2008).

3. Bajic, G., Yatime, L., Sim, R.B., Vorup-Jensen, T. & Andersen, G.R. Structural insight on the recognition of surface-bound opsonins by the integrin I domain of complement receptor 3. *Proc Natl Acad Sci U S A* **110**, 16426-16431 (2013).
