# Supplementary material for: Amyloid-β aggregates activate peripheral monocytes in mild cognitive impairment
Source: Nat Commun. 2024 Feb 9;15:1224. doi: 10.1038/s41467-024-45627-y (PMC10858199; doi:10.1038/s41467-024-45627-y)
Supplement: Supplementary file 3 — Reporting Summary [file 41467_2024_45627_MOESM3_ESM.pdf]

nature portfolio

Corresponding author(s): Thomas Vorup-Jensen

Last updated by author(s): Dec 29, 2023

Reporting Summary

Nature Portfolio wishes to improve the reproducibility of the work that we publish. This form provides structure for consistency and transparency in reporting. For further information on Nature Portfolio policies, see our Editorial Policies and the Editorial Policy Checklist.

Statistics

For all statistical analyses, confirm that the following items are present in the figure legend, table legend, main text, or Methods section.

n/a	Confirmed	
☐	☒	The exact sample size (*n*) for each experimental group/condition, given as a discrete number and unit of measurement
☐	☒	A statement on whether measurements were taken from distinct samples or whether the same sample was measured repeatedly
☐	☒	The statistical test(s) used AND whether they are one- or two-sided<br>*Only common tests should be described solely by name; describe more complex techniques in the Methods section.*
☒	☐	A description of all covariates tested
☐	☒	A description of any assumptions or corrections, such as tests of normality and adjustment for multiple comparisons
☐	☒	A full description of the statistical parameters including central tendency (e.g. means) or other basic estimates (e.g. regression coefficient) AND variation (e.g. standard deviation) or associated estimates of uncertainty (e.g. confidence intervals)
☐	☒	For null hypothesis testing, the test statistic (e.g. *F*, *t*, *r*) with confidence intervals, effect sizes, degrees of freedom and *P* value noted<br>*Give P values as exact values whenever suitable.*
☒	☐	For Bayesian analysis, information on the choice of priors and Markov chain Monte Carlo settings
☒	☐	For hierarchical and complex designs, identification of the appropriate level for tests and full reporting of outcomes
☒	☐	Estimates of effect sizes (e.g. Cohen's *d*, Pearson's *r*), indicating how they were calculated

Our web collection on statistics for biologists contains articles on many of the points above.

Software and code

Policy information about availability of computer code

Data collection	MRI data was collected using commercial scanner (Siemens Skyra 3 T system) and software (Syngo MR v.Ell). PET scans were acquired on a High Resolution Research Tomograph (ECAT HRRT; CTI/Siemens).
Data analysis	For SEM image analysis; ImageJ, version l.52t (NIH) For statistical analysis; Prism, version 9.4.l (GraphPad Software).<br>For mathematical modelling of particle hydrodynamics: Comsol version 6.1, Comsol, Inc.<br>For analysis of fluoresecent-detection mode nanoparticle tracking analysis NanoSight software, version 3.3 and 3.4 with a concentration upgrade (Malvern Panalytical)<br>For analysis of SPR data, EVILFIT version 3 (https://spsrch.cit.nih.gov/software/default.aspx) SeqScape version 2. 7 (Thermofisher)

For manuscripts utilizing custom algorithms or software that are central to the research but not yet described in published literature, software must be made available to editors and reviewers. We strongly encourage code deposition in a community repository (e.g. GitHub). See the Nature Portfolio guidelines for submitting code & software for further information.

Data

Policy information about availability of data

All manuscripts must include a data availability statement. This statement should provide the following information, where applicable:

- Accession codes, unique identifiers, or web links for publicly available datasets
- A description of any restrictions on data availability
- For clinical datasets or third party data, please ensure that the statement adheres to our policy

Raw experimental data from NTA analysis are included in Source Data, including technical replicates for all experiments. All single experiment report summary files generated by NanoSight software for analysis of the MCI cohort are included as supplementary files with an anonymous patient number indicated. Video recordings make up more than 300 GB of data and are available upon reasonable request. SPR raw data is included in the Source Data. The gating strategy for flow cytometry and single stains are included in the Supplementary Section, and raw data are provided with Source Data. Flow cytometry fcs files are available upon reasonable request.
Dataset obtained from public repositories used in the study included structural data with accession numbers #6SHS and #4NEH from the Protein Data Bank (www.rscb.org).

Research involving human participants, their data, or biological material

Policy information about studies with human participants or human data. See also policy information about sex, gender (identity/presentation), and sexual orientation and race, ethnicity and racism.

Reporting on sex and gender	Gender or sex was not used as criteria for collection of patient or healthy control samples. Collected information stratified according to sex was not analyzed as groups were too small to permit such studies.
Reporting on race, ethnicity, or other socially relevant groupings	Information relating to race, ethnicity, or social groupings were not investigated in our study.
Population characteristics	Information on population mean age, sex distribution, and other demographic descriptors are shown in Suppl. Table 1. The APOE genotype of the MCI patients, of relevance for development of Alzheimer's Disease, was analyzed as shown in Suppl. Fig. 6.
Recruitment	MCI subjects were recruited from Dementia/Memory clinics in Jutland and Funen, Denmark, and by newspaper advertisements. Subjects were included if they presented with a history of declining memory function over a minimum of 6 months, preferably corroborated by an informant and in the absence of a history of recreational drug use, sedative medication, depression, stroke or systemic diseases. Further inclusion criteria were: (i) age 50–85 years; (ii) ≥7 years of education or good working history; (iii) meets Petersen criteria (Petersen, 2004) for amnestic MCI (no strict memory score cut-off was used); (iv) an informant was available who had frequent contact with the subject and could accompany the subject to clinic visits or be available to talk on the telephone about the subject's memory and complete the interview for Clinical Dementia Rating (CDR); (v) modified Hachinski Ischaemic Scale score ≤ 4; (vi) Mini-Mental State Examination (MMSE) score 24–30; (vii) Geriatric Depression Scale (GDS-15) score ≤ 6; and (viii) an MRI examination that excluded MCI arising from structural causes.<br><br>Exclusion criteria were: (i) significant neurologic or psychiatric diseases; (ii) history of alcohol and/or recreational drug abuse within 2 years; (iii) contraindications to MRI; (iv) significant reductions in serum B12, red cell folate or thyroid function; and (v) use of medication with known anticholinergic effects (which could impair memory) within the last 3 months or a drug that could impair cognition.<br><br>Age-matched healthy control subjects were recruited by newspaper advertisements and screened for neurological diseases. The same inclusion/exclusion criteria as MCI were applied, except that healthy controls had no complaints of memory decline. In principle, especially the recruitment from newspaper advertisement embodies a risk of self-selection, e.g., by including "worried-well" individuals in the patient group. This risk was reduced by admitting all participants to a clinical dementia rating test before inclusion in the study. The validity of the cohort was confirmed by a CDR global score 0.5. Likewise, the HC group was clinically tested and showed the expected characteristics for HC.
Ethics oversight	The "Central Denmark Region Committees on Health Research Ethics" approved the study Protocol no. 1-10-72-191-14). in accordance with the Declaration of Helsinki. All participants signed an informed written consent at enrollment in the study.<br><br>The use of donor blood for isolation of human monocytes was ethically approved by the Aarhus University Blood Bank (Protocol no. 77).

Note that full information on the approval of the study protocol must also be provided in the manuscript.

Field-specific reporting

Please select the one below that is the best fit for your research. If you are not sure, read the appropriate sections before making your selection.

☒ Life sciences ☐ Behavioural & social sciences ☐ Ecological, evolutionary & environmental sciences

For a reference copy of the document with all sections, see nature.com/documents/nr-reporting-summary-flat.pdf

Life sciences study design

All studies must disclose on these points even when the disclosure is negative.

Sample size	Sample size for mouse experiments was determined in line with 3R principles for limiting the amount of laboratory to a minimum while still obtaining a dataset of sufficient size to rule out the presence of large Aβ aggregates in plasma in line with previously published sample sizes of animal of similar genotype [J Biol Chem. 2008 May 23;283(21):14826-34. doi: 10.1074/jbc.M710574200. Epub 2008 Mar 24.]<br><br>Sample sizes with patients were based on the available MCI-cohort material.<br><br>In vitro experiment samples sizes were based on experience from previous studies published investigating the role of CD18 integrins [J Immunol. 2020 Mar 1;204(5):1345-1361. doi: 10.4049/jimmunol.1900494. Epub 2020 Jan 22].
Data exclusions	No data were excluded.
Replication	All biological experiments were replicated at least once. For experiments using human donor material, the experiments were replicated by analysis of multiple donors, the number of replicate stated in the Figure legends. All attempts to replicate findings were successful as confirmed by relevant statistics were relevant.
Randomization	No randomization was used: all analyses were made using unbiased measuring techniques ensuring an operator-independent result.
Blinding	Only one genotype of mice were used in this study and therefore no blinding was necessary. For the in vitro analyses made, all analyses were made using unbiased measuring techniques requiring no blinding.

Reporting for specific materials, systems and methods

We require information from authors about some types of materials, experimental systems and methods used in many studies. Here, indicate whether each material, system or method listed is relevant to your study. If you are not sure if a list item applies to your research, read the appropriate section before selecting a response.

Materials & experimental systems

n/a	Involved in the study	
☐	☒	Antibodies
☐	☒	Eukaryotic cell lines
☒	☐	Palaeontology and archaeology
☐	☒	Animals and other organisms
☒	☐	Clinical data
☒	☐	Dual use research of concern
☒	☐	Plants

Methods

n/a	Involved in the study	
☒	☐	ChIP-seq
☐	☒	Flow cytometry
☐	☒	MRI-based neuroimaging

Antibodies

Antibodies used

Nanoparticle tracking analysis:
Aducanumab biosimilar, cat. no. PX-TA1335, lot.no. 040721-A01, ProteoGenix
human IgG1,kappa isotype control, cat.no. HG1K, lot.no.MA14OC2803, SinoBiologicals

Integrin function activation, and integrin function blocking:
anti-CD18 clone "KIM127", custom made from American Tissue Culture Collection's hybridoma CRL2838 by GenScript, lot.no. A214111033.
anti-human CD11b clone "ICRF44", cat.no. C0551-50TST, lot.no. 068M4766V, Sigma
anti-human CD11c clone "3.9", cat.no. MAI-46052, lot.no. 0C1681451, ThermoFisher
mouse IgG1 isotype control, clone "MOPC 21", cat.no. M5284, lot.no. 068M4856V, Sigma

Flow cytometry & FACS:
anti-CD11b-PE clone "M1/70", cat.no. 101207, lot.no. B357278, Biolegend
anti-human CD11b-BV421 clone "D12", cat. no. 742637, lot.no. 3114956, BD Optibuild
anti-CD11c-PerCP-eFluor 710 clone "3.9", cat.no. 46-0116-41, lot.no. 2194305, Invitrogen
anti-human-CD14 AF488, clone "63D3", cat.no. 367130, lot.no. B264871, Biolegend
anti-human-CD16 BV605, clone "3G8", cat.no. 563172, lot.no. 7299970, BD Bioscience
anti-human-CD18 BV421, clone "6.7", cat.no. 562871, lot.no. 7223827, BD Bioscience
mouse isotypic IgG1, clone "DAK-GO1", cat. no. X0931, lot.no. 20047017, Agilent

Microscopy
polyclonal anti-human Iba1, cat.no. ab5076. Abcam

rabbit anti-human P2ry12, cat.no.GTX54796, Genetex
secondary Ab donkey anti-goat (Alexa fluor 555), cat.no. A32816, ThermoFisher
donkey anti-rabbit (Alexa Fluor 488), cat. no.711-545-152, Jackson ImmunoResearch

Validation

Nanoparticle tracking analysis:
Aducanumab biosimilar (cat. no. PX-TA1335) and human IgG1,kappa isotype control (cat.no. HG1K) were validated in Suppl. Figure 1 in this study.

Integrin function activation, and integrin function blocking:
KIM127 was validated in Lu et al. (2001) Epitope mapping of antibodies to the C-terminal region of the integrin beta 2 subunit reveals regions that become exposed upon receptor activation. J Immunol. 166:5629-37. doi: 10.4049/jimmunol.166.9.5629.

The following antibodies were validated by the manufacturer as noted below on their indicated website.
anti-human CD11b clone "ICRF44", cat.no. C0551-50TST:https://www.sigmaaldrich.com/DK/en/product/sigma/c0551
anti-human CD11c clone "3.9", cat.no. MAI-46052:https://www.thermofisher.com/antibody/product/CD11c-Antibody-clone-3-9-Monoclonal/MA1-46052
mouse IgG1 isotype control, clone "MOPC 21", cat.no. M5284:https://www.sigmaaldrich.com/DK/en/product/sigma/m5284?gclid=CjwKCAjwrranBhAEEiwAzbhNtTo3fQNoN0hCFYCTEaeDwlJNg-Qhjuxa8JlQlUrEeuJcipw6KsqVmBoCJtkQAvD_BwE

Flow cytometry & FACS:
anti-CD11b-PE clone "M1/70", cat.no. 101207: https://www.biolegend.com/en-us/products/pe-anti-mouse-human-cd11b-antibody-349?GroupID=BLG10552
anti-human CD11b-BV421 clone "D12", cat. no. 742637:https://www.bdbiosciences.com/en-dk/products/reagents/flow-cytometry-reagents/research-reagents/single-color-antibodies-ruo/bv421-mouse-anti-human-cd11b.742637
anti-CD11c-PerCP-eFluor 710 clone "3.9", cat.no. 46-0116-41:https://www.thermofisher.com/antibody/product/CD11c-Antibody-clone-3-9-Monoclonal/46-0116-42
anti-human-CD14 AF488, clone "63D3", cat.no. 367130: https://www.biolegend.com/en-us/clone-search/alexa-fluor-488-anti-human-cd14-antibody-15066?GroupID=BLG14394
anti-human-CD16 BV605, clone "3G8", cat.no. 563172:https://www.bdbiosciences.com/en-us/products/reagents/flow-cytometry-reagents/research-reagents/single-color-antibodies-ruo/bv605-mouse-anti-human-cd16.563172
anti-human-CD18 BV421, clone "6.7", cat.no. 562871: https://www.bdbiosciences.com/en-us/products/reagents/flow-cytometry-reagents/research-reagents/single-color-antibodies-ruo/bv421-mouse-anti-human-cd18.743370
mouse isotypic IgG1, clone "DAK-GO1", cat. no. X0931: https://www.agilent.com/store/en_US/Prod-X093101-2/X093101-2

Microscopy
polyclonal anti-Iba1, cat.no. ab5076:https://www.abcam.com/products/primary-antibodies/iba1-antibody-ab5076.html#:~:text=The%20Iba1%20antibody%20(ab5076)%20is,participates%20in%20membrane%20ruffling%20and
rabbit anti-P2ry12, cat.no.GTX54796: https://www.genetex.com/Product/Detail/P2Y12-antibody/GTX54796
secondary Ab donkey anti-goat (Alexa fluor 555), cat.no. A32816:https://www.thermofisher.com/antibody/product/Donkey-anti-Goat-IgG-H-L-Highly-Cross-Adsorbed-Secondary-Antibody-Polyclonal/A32816
donkey anti-rabbit (Alexa Fluor 488), cat. no.711-545-152, Jackson ImmunoResearch: https://www.jacksonimmuno.com/catalog/products/711-545-152

Eukaryotic cell lines

Policy information about cell lines and Sex and Gender in Research

Cell line source(s)	Human induced pluripotent stem cell (iPSC) line BIHi043A-XM001 [sex: female] and engineered K562 cell lines [sex: female] expressing CR3 and CR4
Authentication	iPSC-cells were tested for sterility, morphology and mycoplasma. Furthermore, the pluripotency was tested with standard markers including NANOG, OCT4, and SOX2. Further information can be found on https://hpscreg.eu/cell-line/BIHi043-A#donor-information. For CR3/K562, CR4/K562 and K562 cell lines, see information collected in Stapulionis et al. (2008) J Immunol. 180:3946-56. doi: 10.4049/jimmunol.180.6.3946. These cell lines were authenticated with regard to their expression of CD11b and CD11c.
Mycoplasma contamination	BIHi043A-XM001 and the K562 cell lines were all tested negative for mycoplasma.
Commonly misidentified lines (See ICLAC register)	K562 and BIHi043A-XM001 cell lines were checked in the ICLAC register with no findings.

Animals and other research organisms

Policy information about studies involving animals; ARRIVE guidelines recommended for reporting animal research, and Sex and Gender in Research

Laboratory animals	All experiments were done with 52 weeks old mice on C57Bl6N background. All test animals lived in standardized conditions where temperature, humidity, and hours of light and darkness are maintained at a constant level all year round. The air is about 22 degrees Celsius; the humidity is about 55 percent; and the light goes on and off automatically, thus imitating a day-night rhythm.
Wild animals	No wild animals were used.
Reporting on sex	Animals of both sexes were taken, each making up 50% of the group. Other than using data from a group of animals with equal sex distribution, sex was not considered in the study, nor was any data disaggregated for sex collected. This descision reflected no priror expectation that sex would form a basis for differences in the experiments made.
Field-collected samples	No field-collected samples were used.
Ethics oversight	All animal experimentation was conducted following approval by local authorities of the Federal State of Berlin (X9007/17).

Note that full information on the approval of the study protocol must also be provided in the manuscript.

Plants

Seed stocks	Not used
Novel plant genotypes	Not used
Authentication	Not used

Flow Cytometry

Plots

Confirm that:

- [x] The axis labels state the marker and fluorochrome used (e.g. CD4-FITC).
- [x] The axis scales are clearly visible. Include numbers along axes only for bottom left plot of group (a 'group' is an analysis of identical markers).
- [x] All plots are contour plots with outliers or pseudocolor plots.
- [x] A numerical value for number of cells or percentage (with statistics) is provided.

Methodology

Sample preparation	Samples were thawed, washed, resuspended in staining buffer, stained with antibodies, and washed again before analysis on the flow cytometer. See Supplementary Materials for further details.
Instrument	For image stream flow cytometry; Amnis ImageStreamX MKII (Amnis). For conventional flow cytometry; Novocyte Analyzer (Acea Biosciences).
Software	For image stream flow cytometry analysis; IDEAS software package (Amnis)<br>For standard flow cytomtry data; FlowJo, version 10.4.2 for PC (BectonDickinson)
Cell population abundance	Cell sorting of microglia was used to separate cells with a low and high abeta uptake. Based on cells single stained with aggregated Hilyte Abeta 488 (AnaSpec cat. AS-60479-01), two limits were set to have 33% of cells in the low Abeta fraction, 33% of cells in the middle, and 33 % of cells with a high Abeta uptake. As a result, samples with a lower abeta uptake would have a shift towards the lower fraction but never below 10 % in the high abeta uptake faction and never below 2,000 events in each gate.

Gating strategy	Events recorded with stable flow were gated by Time vs. CD14 AF488. Live cells were gated as Live-dead nIR neg. events. Monocytes were gated as FSC-high SSC-high events, followed by exclusion of doublet events on a FSC-A vs. FSC-H plot. Lastly, based on expression of CD14 AF488 vs. CD16 BV605 monocytes were subdivided into classical (CD14++ CD16-), intermediate (CD14++ CD16+), non-classical (CD14+ CD16++), and unclassified (CD14- CD16-) monocytes - as shown in Sup. Figure S4.

☒ Tick this box to confirm that a figure exemplifying the gating strategy is provided in the Supplementary Information.

Magnetic resonance imaging

Experimental design

Design type	Resting
Design specifications	1 experiment to collect high-resolution structural images
Behavioral performance measures	No tasks performed

Acquisition

Imaging type(s)	Structural
Field strength	3T
Sequence & imaging parameters	T1-weighted MP2RAGE images were acquired with TR = 5 s, TI1 = 0.7 s, TI2 = 2.5 s, α1 = 4∘, α2 = 5∘, imaged at isotropic 1mm3 resolution (acquisition matrix: 240 × 256, 176 sagittal slices) and turbo factor of 176 as defined by Marques et al. (2010).
Area of acquisition	Whole brain

Diffusion MRI ☐ Used ☒ Not used

Preprocessing

Preprocessing software	MINC - TOOLKIT v2. Non-local means denoising (https://github.com/BIC-MNI/EZminc), brain extraction (https://github.com/BIC-MNI/BEaST)
Normalization	T1-weighted images were spatially normalized to MNI space using both linear and non-linear registrations.
Normalization template	MNI-ICBM152 nonlinear 2009, https://nist.mni.mcgill.ca/atlases/
Noise and artifact removal	Non-local means denoising was used to remove noise in the images.
Volume censoring	Not relevant for the study

Statistical modeling & inference

Model type and settings	Mass univariate mixed effect model.
Effect(s) tested	Not relevant for the study

Specify type of analysis: ☒ Whole brain ☐ ROI-based ☐ Both

Statistic type for inference (See Eklund et al. 2016)	Cluster-wide statistical inference with primary cluster-defining threshold p<0.01.
Correction	FWE

Models & analysis

n/a	Involved in the study
☒	☐ Functional and/or effective connectivity
☒	☐ Graph analysis
☒	☐ Multivariate modeling or predictive analysis
